# Thermoresponsive Polymer Gating System on Mesoporous Shells of Silica Particles Serving as Smart Nanocontainers

**DOI:** 10.3390/polym12040888

**Published:** 2020-04-11

**Authors:** Andrzej Baliś, Karol Wolski, Szczepan Zapotoczny

**Affiliations:** Faculty of Chemistry, Jagiellonian University, Gronostajowa 2, 30-387 Krakow, Poland; balis@chemia.uj.edu.pl (A.B.); wolski@chemia.uj.edu.pl (K.W.)

**Keywords:** core-shell nanoparticles, poly(*N*-isopropylacrylamide), polymer brushes, molecular valves, rhodamine 6G

## Abstract

Spherical silica nanoparticles with solid cores and mesoporous shells (SCMS) were decorated with thermoresponsive polymer brushes that were shown to serve as macromolecular valves to control loading and unloading of a model dye within the mesopores. Thermoresponsive poly(N-isopropylacrylamide) (PNIPAM) brushes were grafted from the surfaces of both solid core (SC) and SCMS particles of similar size using surface-initiated atom transfer radical polymerization. Both systems based on porous (SCMS-PNIPAM) and nonporous (SC-PNIPAM) particles were characterized using cryo-TEM, thermogravimetry and elemental analysis to determine the structure and composition of the decorated nanoparticles. The grafted PNIPAM brushes were found to be responsive to temperature changes enabling temperature-controlled gating of the pores. The processes of loading and unloading in the obtained systems were examined using a model fluorescent dye—rhodamine 6G. Polymer brushes in SCMS-PNIPAM systems were shown to serve as molecular valves enabling significant adsorption (loading) of the dye inside the pores with respect to the SC-PNIPAM (no pores) and SCMS (no valves) systems. The effective unloading of the fluorescent cargo molecules from the decorated nanoparticles was achieved in a water/methanol solution. The obtained SCMS-PNIPAM particles may be used as smart nanocontainers or nanoreactors offering also facile isolation from the suspension due to the presence of dense cores.

## 1. Introduction

Mesoporous silica has been intensively studied and applied since its invention in the 1990s [1,2]. Especially mesoporous silica nanoparticles (MSNs), due to the large surface-to-volume ratio, offer some advantages in many applications. MSNs exhibit an ordered porous structure [3] with high surface area (>1000 m^2^/g) and tunable pore size (2–10 nm) [4,5]. Ordered MSNs are often synthesized using TEOS (tetraetoxysilane) as a silica source and ionic (for e.g., MCM-41 systems) or non-ionic (for SBA-15 systems) surfactants governing inorganic–organic mesophase formation [6]. It has been reported that the size and shape of MSNs can be easily tailored by varying the temperature [7,8], reactants ratio [9], pH [10], surfactant chain length [11], as well as the addition of functional organosilanes [12]. Properly tailored MSNs found applications in numerous fields such as drug delivery systems [13,14], wastewater treatment [15,16], catalysis [17,18], fabrication of nanoreactors [19,20] and nanocontainers [21,22].

There have been numerous examples of nanocarriers composed of thermoresponsive polymers that can be applied for releasing active substances using either temperature-triggered or other types of mechanisms [23,24,25,26,27]. One may observe also a growing interest concerning mechanized silica nanoparticles that utilize supramolecular nanovalves responding to external stimuli for controlled release of encapsulated substances [28,29,30,31,32]. Similar stimuli-responsive functions may be also offered by polymers, especially thermoresponsive ones such as: poly(N-isopropylacrylamide) (PNIPAM) [33,34,35] or poly(*N*-ethyl oxazoline) [36,37] in the form of surface-grafted polymer brushes. PNIPAM chains undergo a phase transition in water from a swollen to collapsed dehydrated state while heating above 32 °C (LCST—lower critical solution temperature) that is related to breaking of hydrogen bonds. These conformational changes of PNIPAM were firstly observed by Heskins and Guillet [38] and later broadly studied and applied both in solutions and for surface grafted macromolecules [39,40]. This behavior of PNIPAM enables the fabrication of various porous and nonporous materials decorated with the polymer chains for e.g., temperature-triggered gating of pores or controlled adsorption/desorption leading to so-called “smart” surfaces [41,42,43,44]. For that purpose typically reversible-deactivation radical polymerizations are used including atom transfer radical polymerization (ATRP) [45] as a primary choice for obtaining not only well-defined macromolecular architectures in solution (e.g., star polymers [46]) but also surface-grafted polymer brushes [47]. While grafting of PNIPAM from silica nanoparticles has been commonly carried out using ATRP [48,49,50] other surface-initiated polymerizations like NMP (nitroxide-mediated polymerization) [51] and ROMP (ring opening metathesis polymerization) [52] have also been applied.

Herein, we present synthesis and temperature-sensitive performance of PNIPAM-gated silica nanoparticles with dense silica cores and mesoporous shells. The presence of dense cores is important for robustness and ease of isolation of the nanoparticles from the aqueous suspension as prerequisites of their applications as reusable nanocontainers and/or nanoreactors. Loading and unloading of a model dye in the mesoporous shells was investigated for various temperatures and solvents to show proper gating mechanism enabling efficient storing but also the release of the encapsulated molecules on demand.

## 2. Materials and Methods

### 2.1. Materials

Tetraethoxysilane (TEOS, 98% GC), hexadecyltrimethylammonium bromide (CTAB, 98%), α-bromoisobutyryl bromide (BIBB, 98%), (3-aminopropyl)triethoxysilane (APTES, >98%), copper (I) bromide, N-isopropylacrylamide (NIPAM, 97%), hydrochloric acid (37%) were purchased from Sigma Aldrich (Darmstadt, Germany). Triethylamine (TEA, >99%), N,N,N′,N″,N″-pentamethyldiethylenetriamine (PMDETA, >99%) were purchased from TCI (Tokyo, Japan). Rhodamine 6G (Rh6G, 99%) was purchased from Acros Organics (Geel, Belgium). Ammonia solution (30%, p.a.), ethanol (96%, p.a.), tetrahydrofuran (THF, p.a.) were purchased from Chempur (Piekary Śląskie, Poland). THF was dried under molecular sieves prior to use. N-isopropylacrylamide was purified by recrystallization from n-hexane. All the other chemicals were used as received. Deionized water was used in all the procedures.

### 2.2. Methods

Thermogravimetric analyses (TG) were performed using a TGA/SDTA 851e Mettler Toledo apparatus (Mettler Toledo, Mississauga, Canada). For the experiments the samples were placed in alumina crucibles and the measurements were carried out in argon (flow 60 mL/min) atmosphere. The samples were heated from 25 to 900 °C with a constant heating rate, β = 10 °C/min (with a 15 min break of heating at 100 °C for each sample). Elemental analyses were performed on a Vario Micro Cube Elemental Microanalyzer (Elementar, Langenselbolt, Germany). Cryogenic Transmission Electron Microscopy images were obtained using a Tecnai F20 TWIN microscope (FEI, Hillsboro, OR, USA) equipped with a field emission gun, operating at an acceleration voltage of 200 kV. Images were recorded using the Eagle 4k HS camera (FEI, Hillsboro, OR, USA) and processed with TIA software (FEI, Hillsboro, OR, USA). Specimen preparation was done by verification of the aqueous solutions on grids with holey carbon film (Quantifoil R 2/2; Quantifoil Micro Tools GmbH, Germany). Prior to use, the grids were activated for 15 s in oxygen plasma using a Femto plasma cleaner (Diener Electronic, Ebhausen, Germany). Cryo samples were prepared by applying a droplet (3 µL) of the solution to the grid, blotting with filter paper and rapid freezing in liquid ethane using a fully automated blotting device Vitrobot Mark IV (FEI, Hillsboro, OR, USA). After preparation, the vitrified specimens were kept under liquid nitrogen until they were inserted into a cryo-TEM-holder Gatan 626 (Gatan Inc., USA) and analyzed in the TEM at −178 °C. FTIR spectra were recorded using a Nicolet iS10 FTIR spectrometer (Thermo Fisher Scientific, Waltham, MA USA) with an ATR accessory. UV-VIS spectra were recorded on a Varian Cary 50 UV-VIS spectrophotometer (Agilent, Santa Clara, CA USA). Scanning electron microscopy studies were performed using a Phenom Pro microscope (Phenom World, Eindhoven, The Netherlands) working at an operational voltage of 10 kV. Silicon substrate was used for the deposition of the obtained particles. The wafer was purified by immersing it in the “piranha” solution (H_2_O_2_/H_2_SO_4_ 1:3 *v*/*v*) for 15 min (Caution! “Piranha” solution is a highly corrosive and oxidative mixture). Afterwards, the coated substrate was washed again with water and the respective suspension of particles was deposited on it by spin-coating (2000 RPM, 1 min). For high-resolution imaging, such prepared dried samples were coated with a nanometric layer of gold. Images of at least 100 particles for each sample with circularity higher than 0.9 were captured at a magnification equal to 40,000× or larger. The diameters of the particles were determined based on the surface areas of the particles from the SEM images using automatic detection offered by Fiji, a Java-based image-processing program developed at the National Institutes of Health.

### 2.3. Procedures

#### 2.3.1. Synthesis of Solid Cores and Mesoporous Shells (SCMS) and Solid Core (SC) Nanoparticles

Solid core mesoporous shell (SCMS) silica nanoparticles were synthesized according to the recently reported two-step procedure [8]. SCMS sample was synthesized at 35 °C (this temperature was kept in both steps). Nonporous solid cores (SC) were synthesized using the same method, but at a lower temperature in order to obtain particles with the average diameters similar to SCMS. The diameters were determined based on SEM images—at least 100 particles were measured and their diameters were averaged.

#### 2.3.2. Synthesis of SCMS-PNIPAM and SC-PNIPAM Nanoparticles

● Preparation of the initiator-coated nanoparticles

**Figure 1:**
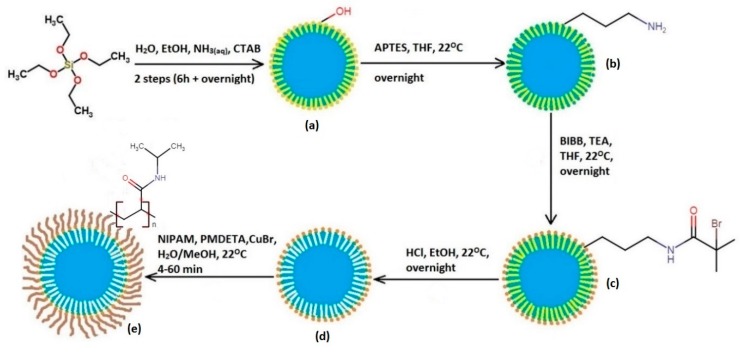
Scheme of the experimental route to obtain solid cores and mesoporous shells (SCMS)- poly(N-isopropylacrylamide) (PNIPAM). Synthesis of: (**a**) SCMS, (**b**) SCMS-APTES, (**c**) SCMS-α-bromoisobutyryl bromide (BIBB), (**d**) SCMS-BIBB(EX), (**e**) SCMS-PNIPAM(xx).

PNIPAM brushes were grafted from the SCMS and SC nanoparticles according to a modified procedure reported by Yang et al. [48] (Figure 1). In the first step an APTES layer was formed on the surface of silica nanoparticles. 2 g of dry SCMS or SC particles was dispersed in 300 mL of anhydrous THF containing 6 mL of APTES. The mixture was sonicated for 15 min, purged with argon for 15 min and then kept overnight at room temperature. The final products (SCMS-APTES and SC-APTES) were centrifuged, washed with methanol (shaking and sonication) and finally dried under vacuum. Then, BIBB was grafted onto the surface of the amine-group functionalized SCMS-APTES and SC-APTES. An amount of 1.745 g of each nanoparticle sample was dispersed in the mixture of 300 mL of dry THF and 15 mL of TEA that was sonicated for 15 min and purged with argon for 15 min. Then 4.2 mL of BIBB was added dropwise to the dispersion that was left overnight at room temperature. The obtained mixture was added to 1 L of deionized water and vigorously stirred for 1 h. Such obtained products (SCMS-BIBB and SC-BIBB) were separated via sedimentation. The modified particles were washed with acetone and toluene repeatedly and dried under vacuum.

● Extraction of CTAB template

CTAB surfactant templates were removed from porous structures of SCMS-BIBB by mixing 1.1 g of the sample with acidic ethanol solution (2.5 mL of 37% HCl in 250 mL of ethanol) overnight at room temperature. Non-porous SC-BIBB did not undergo this procedure.

● Grafting of PNIPAM brushes

PNIPAM brushes were grafted from SCMS and SC via the SI-ATRP method based on the procedure previously reported by us [53]. The reaction system was composed of four conical flasks sealed with rubber septa and connected with double-tipped needles to transfer the monomer solution under argon atmosphere. Solvent mixture (methanol/water: 4/1 *v*/*v*, total volume 30 mL) was placed in the first flask in order to saturate the whole reaction system with its vapors. The monomer mixture in the second flask containing NIPAM (7.14 g) and PMDETA (1 mL) was diluted in 28.5 mL of methanol and 7.2 mL of water. In the third flask, CuBr (95 mg) and a magnetic stirring bar were placed. SC or SCMS (each 0.86 g) were placed in the fourth flask equipped with a magnetic stirring bar and dispersed in 9.5 mL of methanol and 2.4 mL of water by sonication prior to the polymerization. The system was then purged with argon for 15 min. The degassed mixture of the monomer and ligand from the second flask was transferred via double tipped needle in the flow of argon to the third flask with CuBr. The mixture was stirred at ambient temperature for 20 min to dissolve the CuBr. The obtained solution was then transferred to the last flask with SC and SCMS to start the polymerization. Five fractions were collected after 4, 8, 12, 20, 60 min via syringe with a thick needle. All fractions were transferred to the separated vials and an excess of the methanol was quickly added to each vial in order to stop the polymerization. The resulting products were purified from the unbound free polymer and residual salts by washing four times with cold water (1 cycle: sonication 10 min, shaking 10 min, centrifugation 5 min, 7900 RCF). The final products named SC-PNIPAM(xx) and SCMS-PNIPAM(xx) (where xx is the polymerization time in minutes) were stored in aqueous solution in a fridge.

#### 2.3.3. Adsorption Studies of Rhodamine 6G

For the adsorption studies ca. 10 mg of a given sample (SCMS-PNIPAM(xx), SC-PNIPAM(xx) or extracted SCMS) was dispersed in 2 mL of water. Afterwards 10 mL of Rh6G aqueous solution (c = 5 mg/L) was added and shaken for 1 h using vortex at 22 or 50 °C. Afterwards, the suspension was cooled down to 22 °C and centrifuged (5 min, 7900 RCF). UV-VIS spectra of the supernatant were measured.

#### 2.3.4. Releasing of Rhodamine 6G from Nanoparticles

5 samples of dry SCMS-PNIPAM (4) (ca. 5 mg each) were dispersed in 1 mL of water by sonication (5 min). 5 mL or Rh6G solution (c = 5 mg/L) was added to each dispersion that was then shaken for 1 h with vortex at 50 °C. Afterwards, the dispersion was cooled down to close the pores (5 min in a beaker with ice and water) centrifuged (5 min, 7900 RCF) and UV-VIS spectrum of the supernatant was measured. Then, each sample was dried overnight and then 6 mL of water/methanol mixtures of various methanol contents (methanol molar fractions, x_MeOH,_ equal to: 0, 0.05, 0.2, 0.45, 1) was added to the samples and shaken for 1 h at room temperature. Finally, the samples were centrifuged and UV-VIS spectra were measured.

#### 2.3.5. Determination of the Lower Critical Solution Temperature (LCST) Value Using Dynamic Light Scattering (DLS)

DLS measurements of the aqueous dispersion of SCMS-PNIPAM particles were performed using a Malvern Nano ZS instrument working at 173° detection angle, at various temperatures in the range 22–45 °C. The measured hydrodynamic diameters were averaged from three parallel measurements with 10–100 runs each for every temperature.

## 3. Results

The silica nanoparticles (average diameter equal to 380 ± 30 nm; see Figure S1 for representative SEM images) with solid cores and porous shells decorated with thermosensitive PNIPAM brushes (SCMS-PNIPAM) of various thicknesses were synthesized and studied. For comparison, nonporous silica particles of similar diameter (SC-PNIPAM) were obtained and temperature-dependent adsorption and desorption of a model dye (Rh6G) was studied for both systems.

### 3.1. Characterization of SC-PNIPAM and SCMS-PNIPAM

#### 3.1.1. FTIR Spectra

Brush-decorated nanoparticles, SC-PNIPAM and SCMS-PNIPAM and appropriate intermediate products, were characterized by FTIR spectroscopy (Figures S2 and S3). All the spectra of the materials based on nonporous, SC, nanoparticles are dominated by very intensive bands with the maximum at 1040 cm^−1^, characteristic of silicon oxide groups (Figure S2). The band at 3422 cm^−1^ in SC and SCMS spectra corresponds to -OH stretching vibrations while the band at 1634 cm^−1^ may be assigned to residual water. Deposition of the APTES monolayer did not cause any significant changes in the spectrum due to the small amount of APTES molecules with respect to the silica material. After grafting of BIBB initiator new bands assigned to C–H stretching vibrations appeared in the range 2900–3000 cm^−1^ (Figure S2). The FTIR spectrum of the sample after polymerization shows characteristic bands for PNIPAM such as: amide I (C=O stretching vibrations) at 1639 cm^−1^ and amide II (deformation vibrations of N–H) at 1534 cm^−1^. The spectra of the samples based on SCMS nanoparticles are presented in Figure S3. Strong and sharp bands at 2853 and 2923 cm^−1^ in the spectrum of native SCMS can be assigned to the anti-symmetrical and symmetrical stretching vibrations of –CH_2_– in CTAB surfactant molecules while the band at ca. 1475 cm^−1^ corresponds to the deformation vibration of –NH_3_^+^ and –CH_2_–. These bands diminished after extraction of the CTAB template (SCMS-BIBB(EX)). After polymerization, characteristic amide I and II vibrations from PNIPAM appeared in the FTIR spectrum proving the successful growth of the brushes on the SCMS surface (SCMS-PNIPAM).

#### 3.1.2. Thermogravimetric Analysis

**Figure 2:**
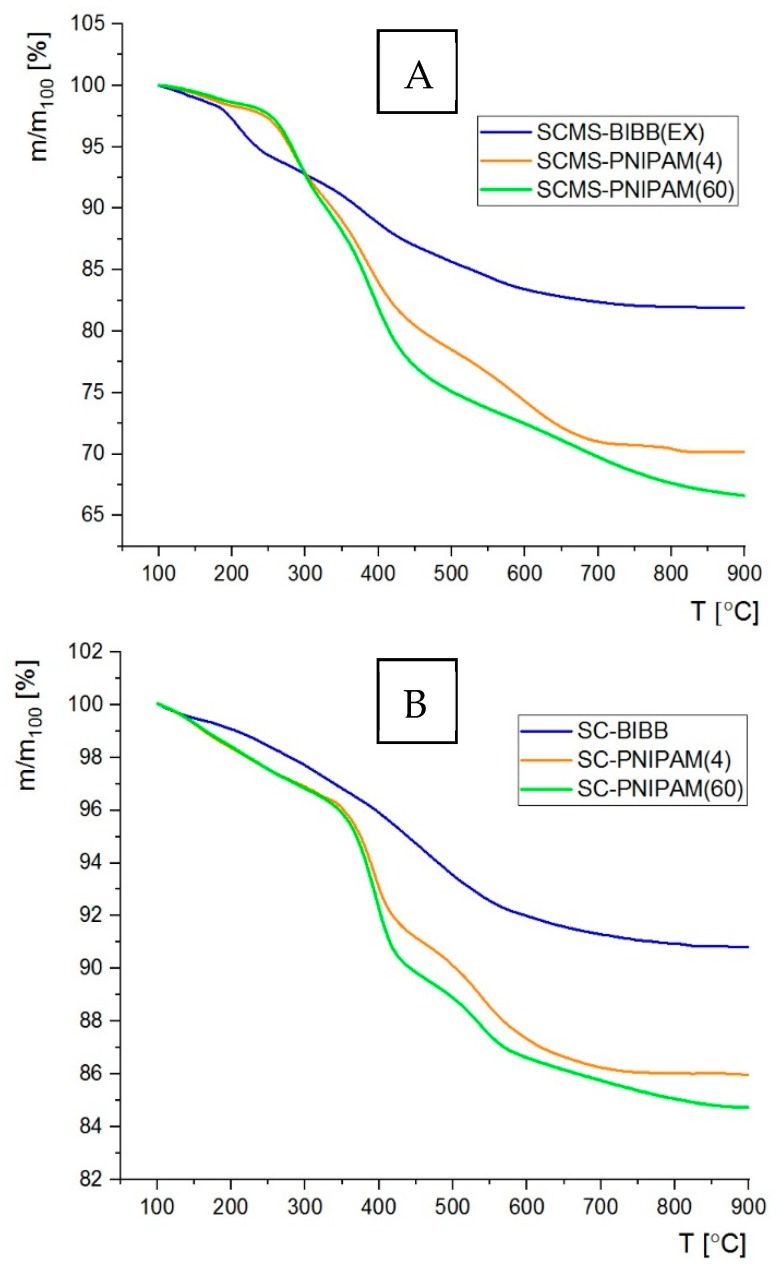
Thermogravimetric analyses of the samples based on (**A**) SCMS, and (**B**) SC particles.

The results of TGA of the SC-PNIPAM and SCMS-PNIPAM are plotted in Figure 2A,B. The samples of SC and SCMS before and after polymerization (4 and 60 min) were studied (see further details for the reasons behind this selection). The total weight loss of BiBB-modified SC and SCMS reached, respectively, 8 wt% and 15 wt% proving successful grafting of the initiator. The difference can be related to the residual surfactant that remained in the pores of SCMS-BIBB sample or deposition of BIBB also partially in the pores. Importantly, the template molecules were intentionally removed only after deposition of the initiator molecules in order to spatially limit subsequent growth of PNIPAM to the very surface of the nanoparticles (not within the pores) as otherwise they could have been permanently closed. However, some adsorption of APTES at the opening of the pores cannot be excluded. One cannot neglect also the much smaller specific density of SCMS vs. SC that implies larger relative mass content of the adsorbed BIBB for the former nanoparticles. The total weight loss in the brush-decorated samples was 14–15 wt% for SC-PNIPAM and 29–33 wt% for SCMS-PNIPAM. The difference can be related to smaller specific density of the initial SCMS leading to increased contribution of the polymer mass in total weight of SCMS-PNIPAM and also to a partial grafting of PNIPAM inside the pores. Moreover, the slope of the TGA curve of SC-PNIPAM was found to change between 330 °C and 430 °C pointing to thermal degradation of PNIPAM in this temperature range. In the porous SCMS such a decrease occurs in a broader temperature range. It starts at 250 °C and finishes around 430 °C. It is probably related to residual CTAB and/or BIBB molecules which are not totally removed during the extraction process (CTAB decomposes below 300 °C) [54].

#### 3.1.3. Elemental Analysis

Elemental composition of the obtained materials was determined via elemental analysis and plotted in Figure S4 (SC) and Figure S5 (SCMS). The percentage of carbon and nitrogen was measured for several fractions after various polymerization times (after 4, 8, 12, 20, 60 min) in order to estimate the amount of PNIPAM in the studied samples. As shown in Figures S2 and S3, carbon and nitrogen content reached an almost constant value after only 4 min of polymerization, indicating a very fast process. Only minor changes in the composition were observed between 4 and 60 min. It can be attributed to rapid generation of the deactivator (CuBr_2_) due to the high concentration of the growing macroradicals in the dispersion of the nanoparticles and/or irreversible termination of some of the growing macroradicals that slows down the polymerization [34]. Thus, for further adsorption studies only the samples after 4 and 60 min of PNIPAM polymerization were chosen—SCMS-PNIPAM(4) and SCMS-PNIPAM(60), respectively. Approximately twice the level of carbon and nitrogen content in decorated SCMS compared to SC nanoparticles can be rationalized using similar reasoning to that used for discussing the thermogravimetric results.

#### 3.1.4. Cryo-TEM Imaging

**Figure 3:**
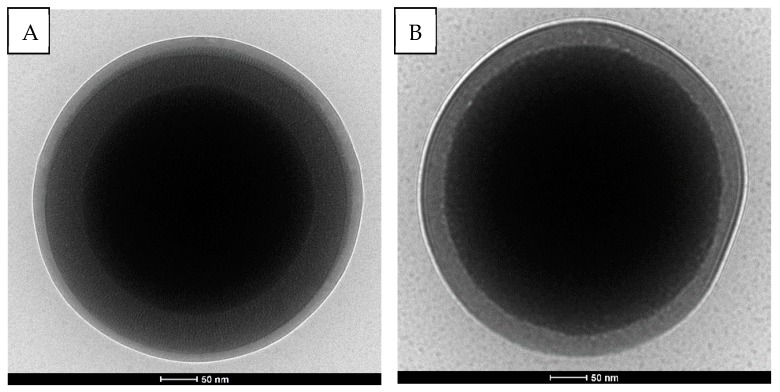
Cryo-TEM images of (**A**) SCMS-PNIPAM(4), and (**B**) SC-PNIPAM(4).

TEM working in cryogenic mode was used to determine morphology changes in the obtained silica-polymer systems. PNIPAM brushes in SC-PNIPAM(4) was found to be approximately 35 nm thick while in SCMS-PNIPAM(4) it was around 20 nm (Figure 3). Thicker brushes on SC seem to be related to the higher grafting density of the PNIPAM with respect to SCMS that is easier to obtain on smooth (SC) rather than rough and porous surfaces (SCMS). As a reference, cryo-TEM images of raw SCMS and SC nanoparticles are shown in Figure S6. The influence of temperature on the conformation of PNIPAM brushes was also tested using cryo-TEM methods (Figure S7). Suspension of the sample polymerized for 20 min was heated to 50 °C before the measurement. It appears that the structure of the PNIPAM brushes changes by increasing the temperature above the LCST due to repealing of water molecules from the brushes. However, the overall thickness of the PNIPAM layer does not significantly decrease after heating likely due to vertical but not horizontal aggregation of the chains as reported previously for flat substrates [35]. The observed bubble-like structures within the brushes (Figure S7A) indicate the presence of caged water/air above the LCST. Partial horizontal collapse of the PNIPAM chains cannot be excluded, but detailed behavior of such systems with respect to molar mass of PNIPAM, grafting density, the surface curvature and roughness is still under debate and its elaboration is beyond the scope of the current report [42]. Nevertheless, the postulated aggregation of the neighboring elongated chains (no total collapse of the chains) is advantageous for the intended gating applications since the collapsed chains could have formed a dense and continuous layer blocking the openings of the pores. The LCST value for the PNIPAM chains grafted on SCMS particles was also determined using the measurements of the hydrodynamic diameters of SCMS-PNIPAM(4) via DLS in the function of temperature (Figure S8). The observed decrease of the hydrodynamic diameter should be correlated with the changes of the conformation of the PNIPAM chains at LCST that was determined to be equal to 32 °C from the inflection point of the sigmoidal relationship. The value is in very good agreements with the LCST value measured for free LCST chains in water.

### 3.2. Adsorption of Rh6G within SCMS-PNIPAM and SC-PNIPAM Particles

#### 3.2.1. Adsorption of the Rh6G Dye

**Figure 4:**
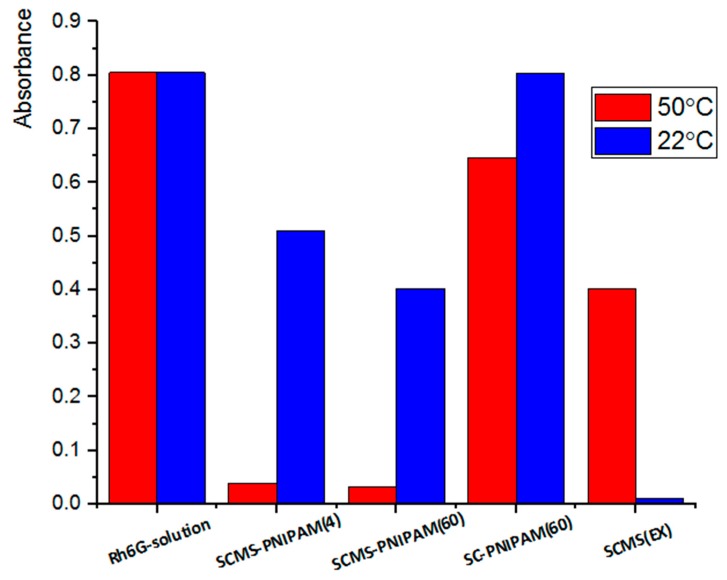
Adsorption of model rhodamine 6G (Rh6G) dye from its solution by various nanoparticles systems as measured by UV/Vis absorption of the supernatant at λ = 527 nm (max. absorbance of Rh6G).

The results of the adsorptions studies of a model Rh6G dye within both porous and nonporous, systems are presented in Figure 4. All the samples were immersed in the Rh6G solution of the same concentration (UV/Vis absorbance) and the decrease of this concentration due to absorbance of the dye molecules was measured spectrophotometrically. The lower the absorbance of the supernatant the larger the amount of the adsorbed dye. It can be noticed that the dye was the most efficiently adsorbed by the SCMS-PNIPAM systems at 50 °C (concentration was reduced by more than 95%) when the gating PNIPAM brushes were aggregated enabling migration of the dye molecules into the pores. Subsequent cooling of the systems closed the pores so the dye molecules could not leak from such nanocontainers. If the adsorption was carried out at 22 °C (below LCST) the PNIPAM chains became extended and swollen closing the openings of the pores. It results in a much smaller reduction of the dye concentration in the supernatant: by ca. 37% for SCMS-PNIPAM(4) and ca. 50% for SCMS-PNIPAM(60). A similarly decorated but nonporous SC-PNIPAM(60) system exhibited poor adsorption ability at both temperatures (20% at 50 °C and only 1% at 22 °C) indicating that the adsorption takes place mostly in the pores if they are available in the nanoparticles but not within the brushes. No substantial differences in the dye adsorption ability could be observed between the SCMS-PNIPAM(4) and SCMS-PNIPAM(60) pointing to formation of a sufficiently thick PNIPAM shell after only 4 min of polymerization. Importantly, at room temperature migration of Rh6G to the negatively charged surface of silica pores is blocked by the swollen PNIPAM brushes, whereas the adsorption ability of uncoated SCMS(EX) is more effective at a lower rather than higher temperature. Thus, the dependence of adsorption of Rh6G on temperature is opposite for the native and decorated SCMS nanoparticles with thermoresponsive brushes. The observed difference should be attributed to the presence of PNIPAM brushes on the surface of SCMS that even for a collapsed conformation at 50 °C may cause narrowing of the pores’ openings. This way cylindrical pores are converted into bottle-like pores (diameter of the opening is smaller than the diameter of the pore) characterized of significant adsorption–desorption hysteresis [55]. Thus, in the case of SCMS-PNIPAM, the dye molecules, once transported into and adsorbed on the oppositely charged pores’ surface, cannot rapidly diffuse out implying the high adsorption capacity of the system. For SCMS with cylindrical pores the adsorption/desorption equilibrium is reached faster so the measured adsorption of the dye decreases with the increase in temperature as it can be expected.

#### 3.2.2. Release of Cargo Molecules to Water/Methanol Mixture

**Figure 5:**
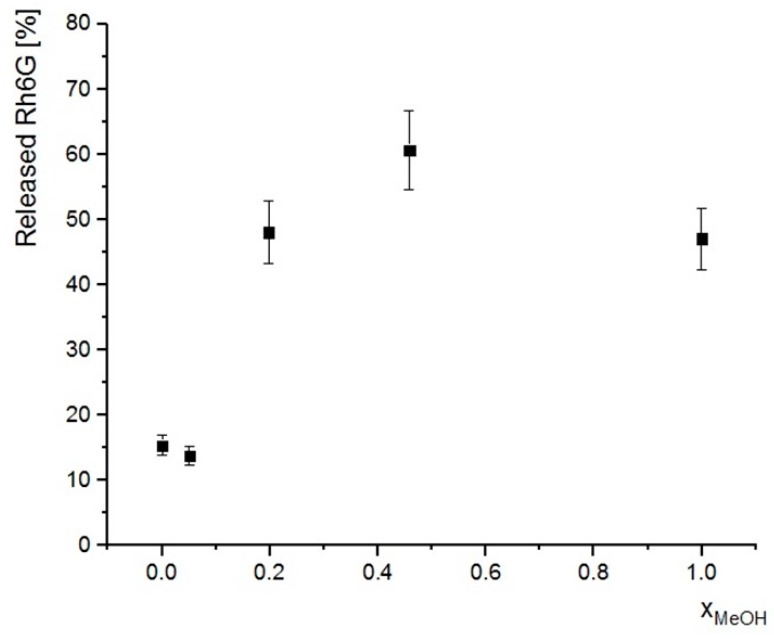
Release of Rh6G adsorbed in SCMS-PNIPAM(4) to water/methanol mixtures of various methanol contents (x_MeOH_). The amount of the released dye (determined spectrophotometrically) is presented as percentage of the total adsorbed amount.

The release of the adsorbed Rh6G from the nanoparticles to pure water above LCST was found inefficient confirming the strong adsorption of the positively charged dye molecules on the negatively charged silica surface. Thus, the release of the cargo molecules was achieved in a water/methanol mixture. While the presence of methanol should promote desorption of Rh6G, PNIPAM chains undergo also reversible “coil-globule” transition at certain compositions of water/alcohol mixtures as shown both in solution [56] and for polymer brushes [33,57]. Figure 5 presents the efficiency of release of the cargo Rh6G molecules from the previously loaded SCMS-PNIPAM(4). One may expect more efficient release of Rh6G for the increasing content of methanol but it appears that the relation in Figure 5 shows a clear maximum. A low concentration of methanol in the mixture does not lead to collapse of the PNIPAM chains so the release efficiency is practically the same as for pure water. Only after increasing the methanol content (x_MeOH_ = 0.2) the PNIPAM chains aggregate opening the pores and the release efficiency increases significantly to ca. 50%. A further increase of methanol content to x_MeOH_ equal to ca. 0.45 enhances the release of the dye. Lower efficiency was obtained for pure methanol, which on one side enhances the desorption of the dye, but on the other side increases the swelling of PNIPAM chains that limit diffusion of Rh6G from the pores. This is consistent with the co-nonsolvency effect—PNIPAM brushes become soluble in the mixture with x_MeOH_ > 0.5 [58]. Thus, although more methanol in the mixture should enable more efficient desorption of Rh6G molecules the gating system limits their diffusion from the pores.

## 4. Conclusions

The spherical silica nanoparticles (average diameter 380 ± 30 nm) with solid cores and mesoporous shells (SCMS) decorated with thermosensitive PNIPAM brushes serving as molecular valves were fabricated and their performance was studied. The template surfactant molecules were extracted from the formed SCMS particles after surface deposition of the ATRP initiators in order to limit the subsequent growth of the brushes only to the surface of the nanoparticles but not the interior of the pores. The SCMS-PNIPAM particles obtained after various polymerization times were characterized using FTIR spectroscopy, thermogravimetry, elemental analysis as well as cryo-TEM. It was shown that the surface-initiated ATRP at the studied conditions proceeds very fast at the beginning and slows down likely due to rapid generation of the deactivator (CuBr_2_) as a result of a high concentration of the growing macroradicals on the surface of the suspended nanoparticles and/or their irreversible termination. Importantly for the gating applications of the PNIPAM brushes, the cryo-TEM imaging of the system heated to the temperature above LCST indicated vertical aggregation of such densely grafted brushes rather than collapsing of the chains that otherwise could have totally blocked the pores at higher temperatures. Thus, it was shown that the studied SCMS-PNIPAM systems can very efficiently adsorb model rhodamine 6G dye molecules within the mesopores even at 50 °C that was not observed for undecorated particles at the same temperature. Such loaded systems were also shown to be unloaded by dispersion of them in a water/methanol mixture. The most efficient release of the dye was shown for x_MeOH_ = 0.45 while in pure methanol the efficiency was significantly lower. This is consistent with the co-nonsolvency effect for PNIPAM—the brushes become aggregated for medium content of methanol (x_MeOH_ in the range ca. 0.15 to 0.5) while they are well-swollen for both low and high methanol content. Thus, the fabricated silica core-shell nanoparticles with thermosensitive polymer brushes grafted from their surface were shown to serve as nanocontainers with high adsorption capabilities even at elevated temperatures and tunable unloading performance by varying methanol content. Thanks to the presence of solid cores the nanoparticles were mechanically robust and not susceptible to destruction during the repeated separation processes by centrifugation; this is an important feature for their possible applications.

## Supplementary Materials

The following are available online at https://www.mdpi.com/2073-4360/12/4/888/s1. Figure S1. Representative SEM image of (A) SC, (B) SCMS nanoparticles; Figure S2. (A) FTIR spectra of SC-PNIPAM and the intermediate products, (B) zoomed-in spectra in the range 1300 to 3100 cm^−1^; Figure S3. (A) FTIR spectra of SCMS-PNIPAM and the intermediate products, (B) zoomed-in spectra in the range 1300 to 3100 cm^−1^; Figure S4. Results of elemental analysis (carbon and nitrogen) of SC-PNIPAM samples after various polymerization times; Figure S5. Results of elemental analysis (carbon and nitrogen) of SCMS-PNIPAM samples after various polymerization times; Figure S6. Cryo-TEM images of (A) SC and (B) SCMS nanoparticles; Figure S7. Cryo-TEM image of (A) SC-PNIPAM(20)H (heated to 50 °C before the measurement) and (B) SC-PNIPAM(20) prepared at room temperature; Figure S8. Changes of the hydrodynamic diameter (by DLS) of SCMS-PNIPAM(4) dispersed in water at various temperatures. The solid line represents the sigmoidal fit to the obtained data and the vertical dotted line indicates the inflection point at 32.0 °C.
