# Supplementary material for: Thermoresponsive Polymer Gating System on Mesoporous Shells of Silica Particles Serving as Smart Nanocontainers
Source: Polymers (Basel). 2020 Apr 11;12(4):888. doi: 10.3390/polym12040888 (PMC7240617; doi:10.3390/polym12040888)
Supplement: Supplementary file 1 [file polymers-12-00888-s001.pdf]

Supplementary Materials

Thermoresponsive polymer gating system on mesoporous shells of silica particles serving as smart nanocontainers

Andrzej Baliś, Karol Wolski, Szczepan Zapotoczny

Jagiellonian University, Faculty of Chemistry, Gronostajowa 2, 30-387 Krakow, Poland

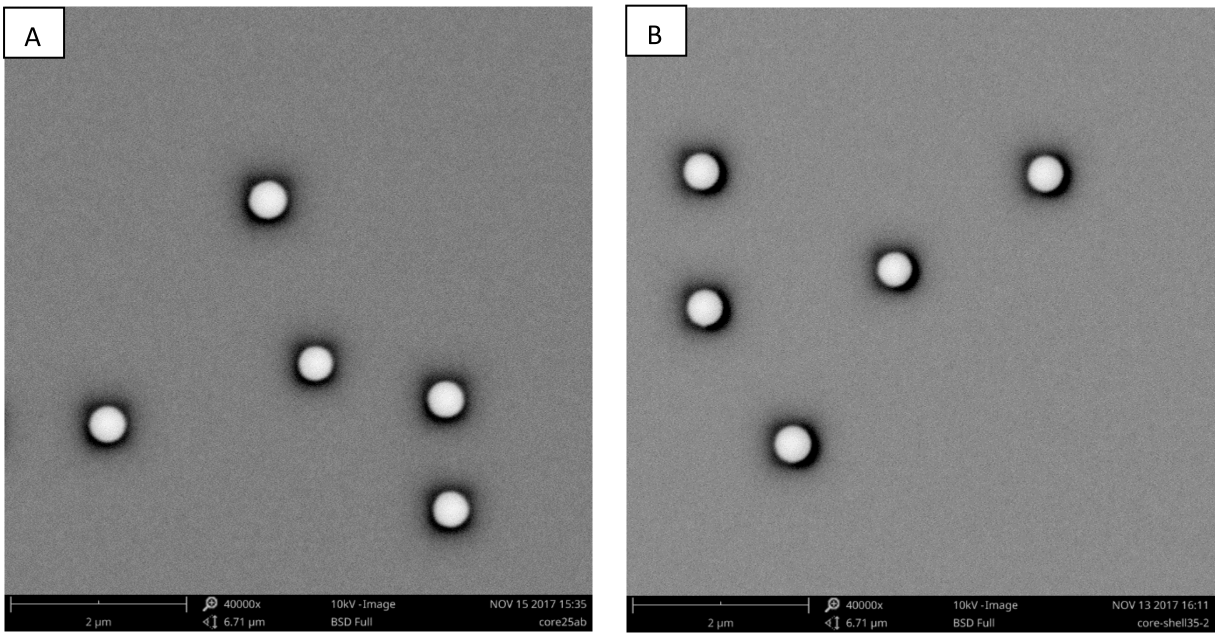

Figure S1. Representative SEM image of (A) SC, (B) SCMS nanoparticles.

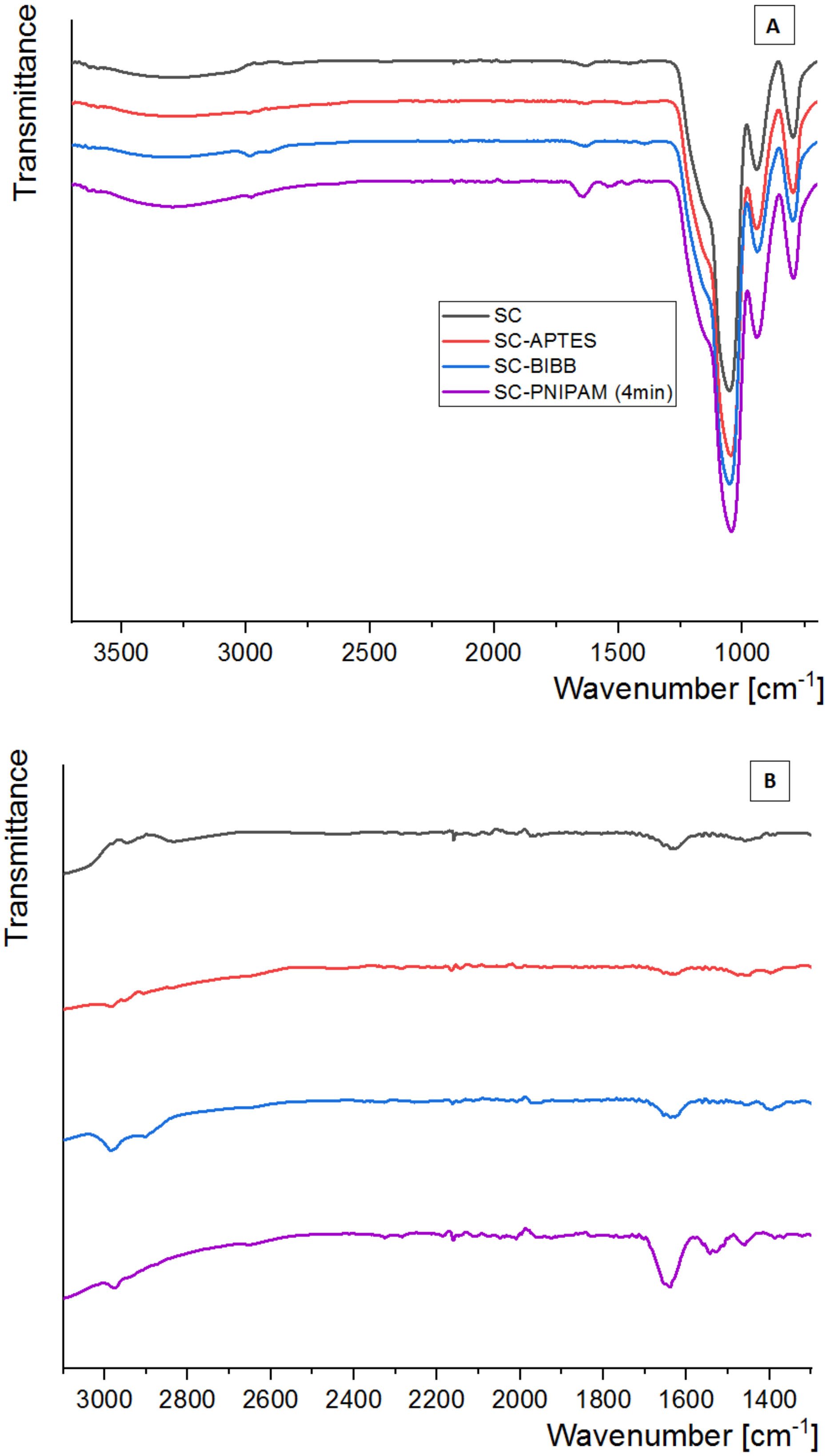

Figure S2. (A) FTIR spectra of SC-PNIPAM and the intermediate products, (B) zoomed-in spectra in the range 1300 to 3100cm^{-1}.

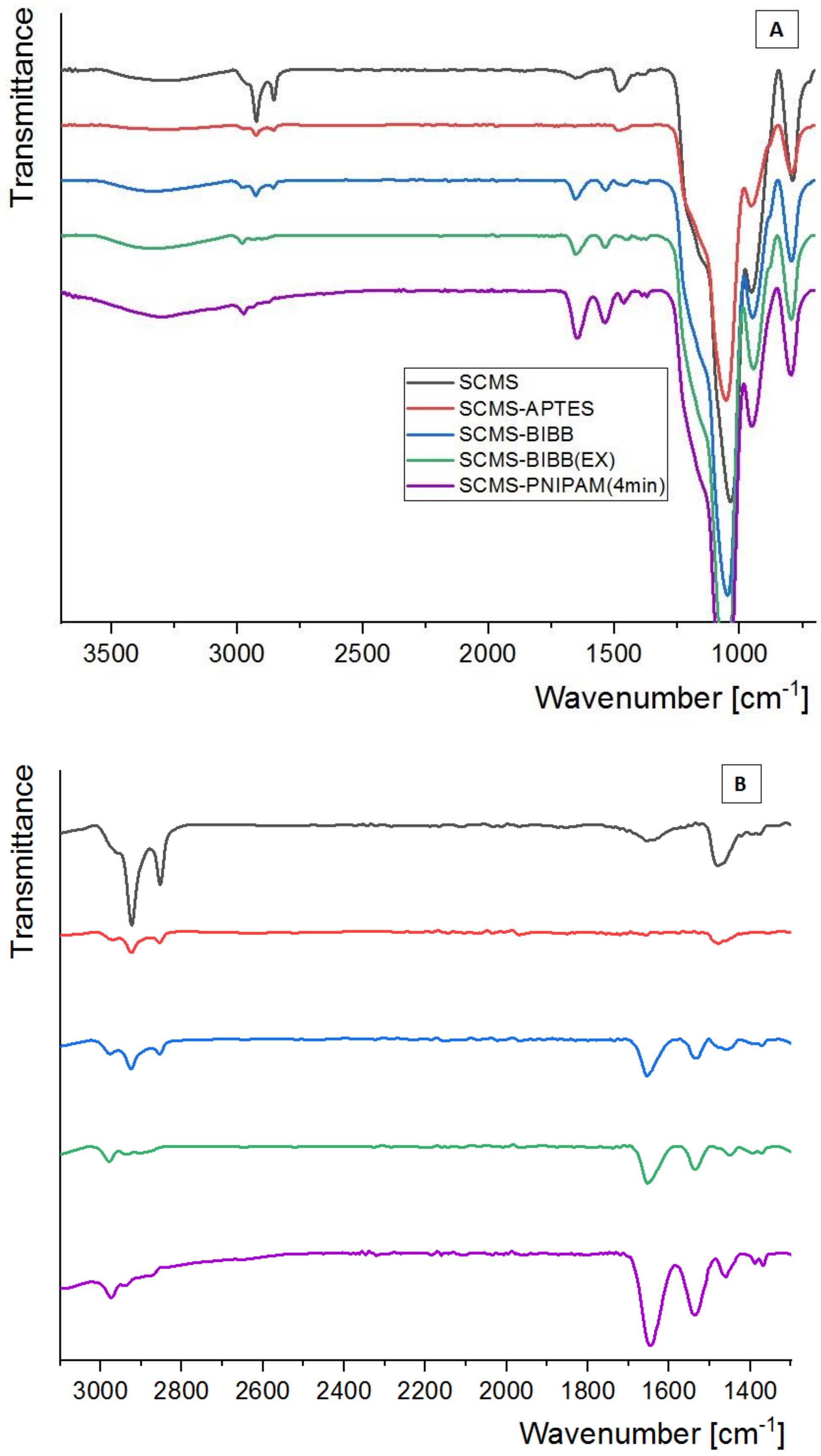

Figure S3. (A) FTIR spectra of SCMS-PNIPAM and the intermediate products, (B) zoomed-in spectra in the range 1300 to 3100cm^{-1}.

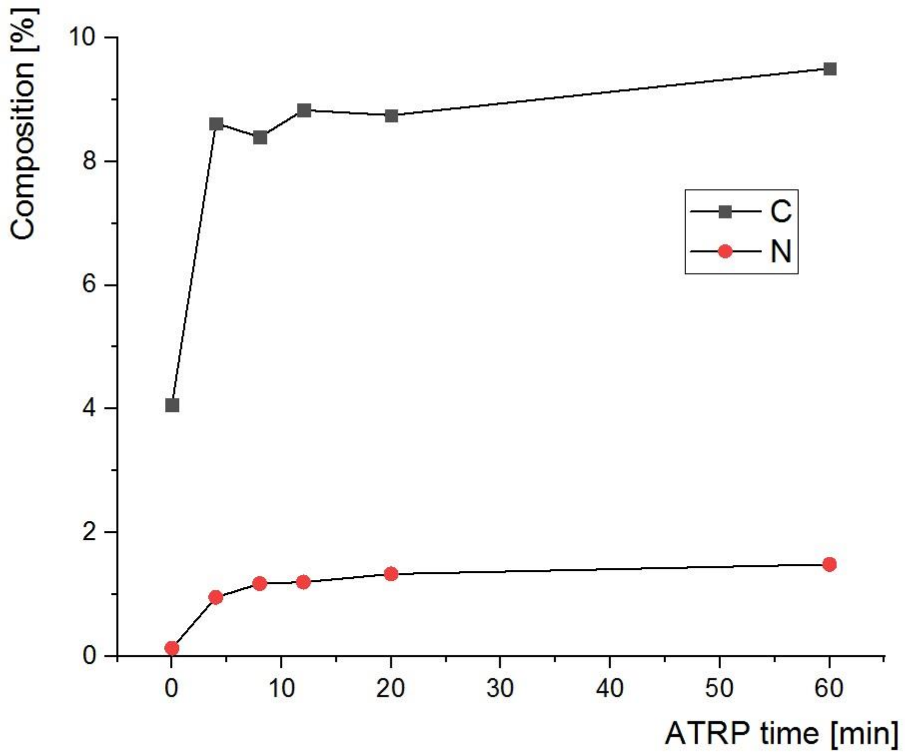

Figure S4. Results of elemental analysis (carbon and nitrogen) of SC-PNIPAM samples after various polymerization times.

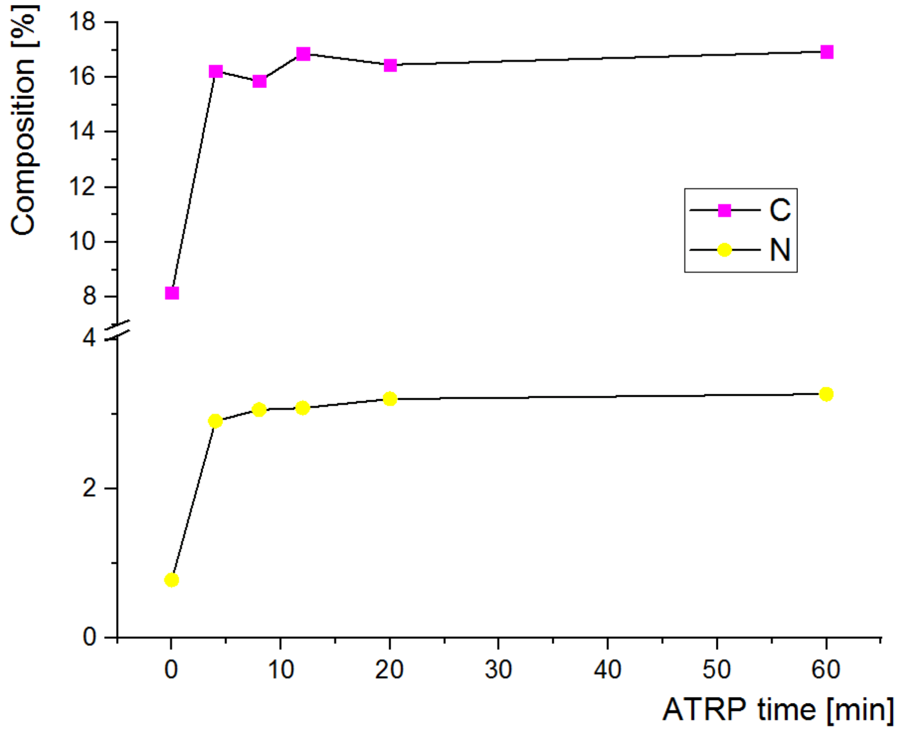

Figure S5. Results of elemental analysis (carbon and nitrogen) of SCMS-PNIPAM samples after various polymerization times.

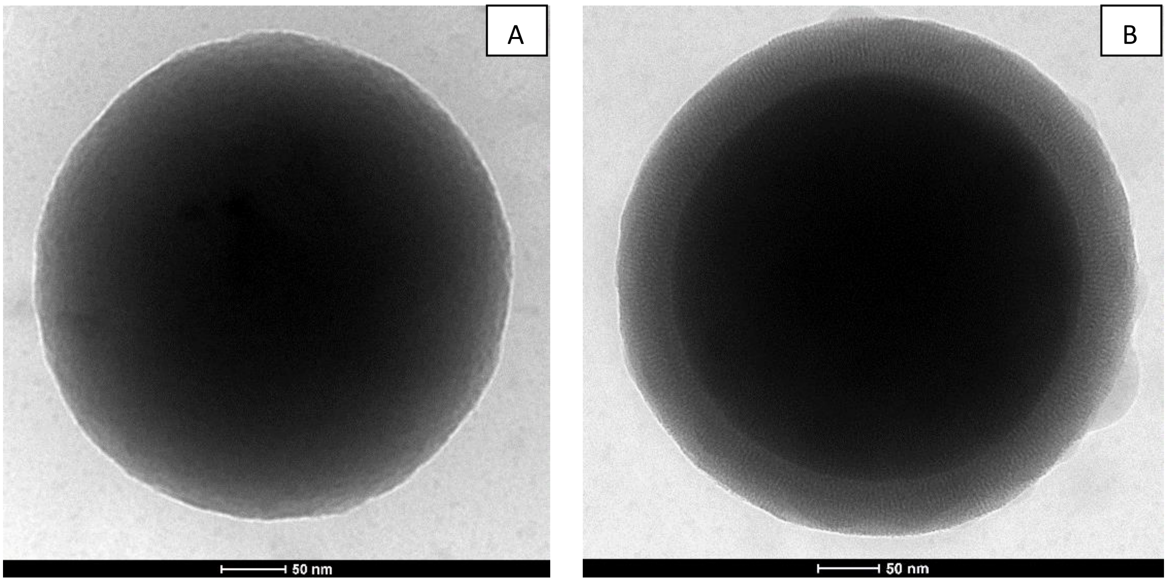

Figure S6. Cryo-TEM images of (A) SC and (B) SCMS nanoparticles.

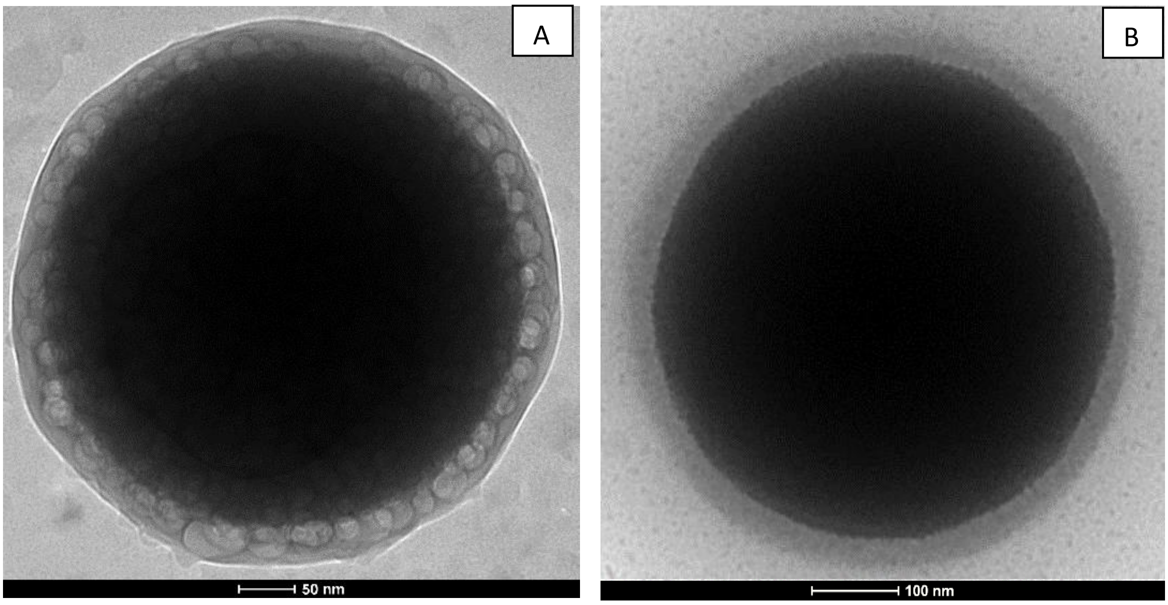

Figure S7. Cryo-TEM image of (A) SC-PNIPAM(20)H (heated to 50°C before the measurement) and (B) SC-PNIPAM(20) prepared at room temperature.

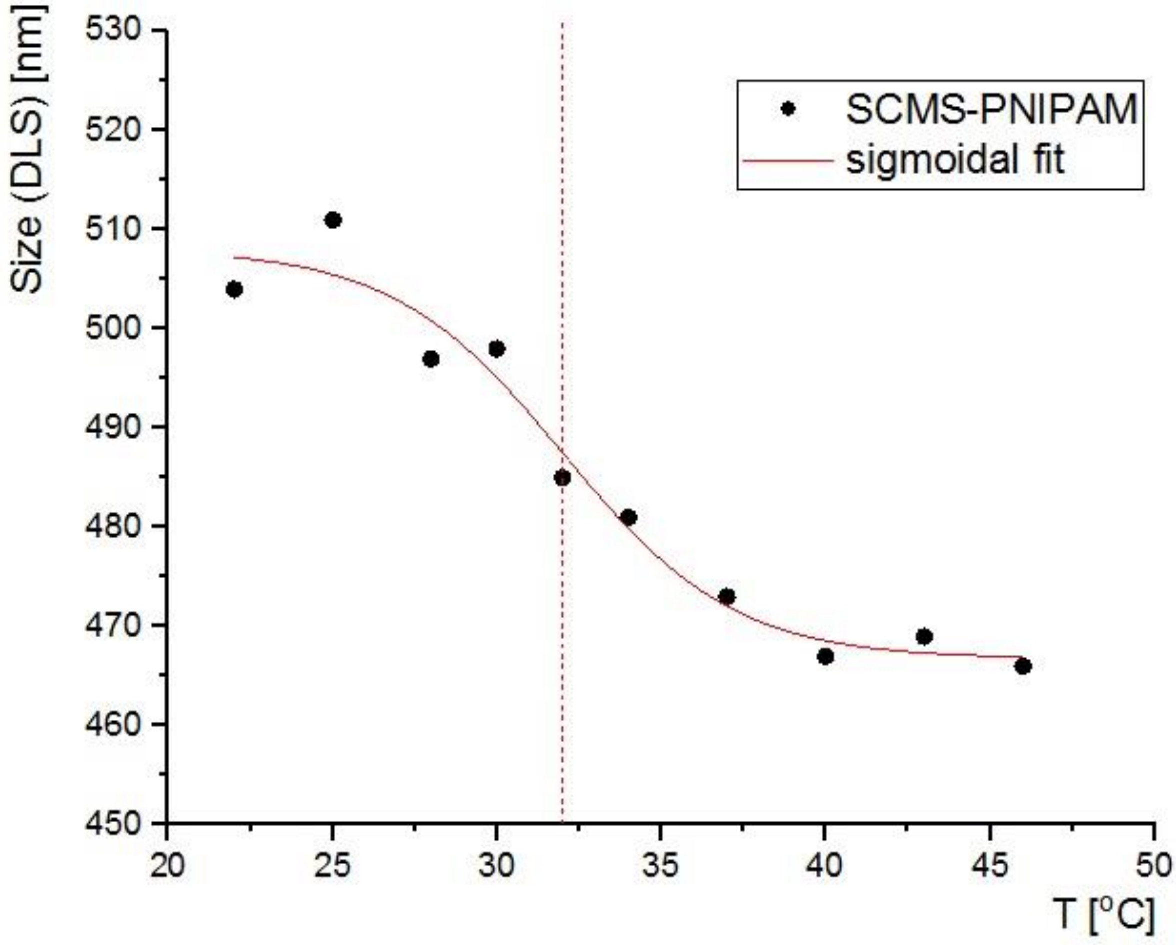

Figure S8. Changes of the hydrodynamic diameter (by DLS) of SCMS-PNIPAM(4) dispersed in water at various temperatures. The solid line represents the sigmoidal fit to the obtained data and the vertical dotted line indicates the inflection point at 32.0°C.
